# Screening Methods to Discover the FDA-Approved Cancer Drug Encorafenib as Optimally Selective for Metallothionein Gene Loss Ovarian Cancer

**DOI:** 10.3390/genes16010042

**Published:** 2025-01-01

**Authors:** Amy Rees, Evan Villamor, Della Evans, Monika Gooz, Clare Fallon, Mirna Mina-Abouda, Andrew Disharoon, Scott T. Eblen, Joe R. Delaney

**Affiliations:** 1 Department of Biochemistry and Molecular Biology, Medical University of South Carolina, Charleston, SC 29425, USA; 2 Department of Drug Discovery and Biomedical Sciences, Medical University of South Carolina, Charleston, SC 29425, USA; 3 Department of Pharmacology and Immunology, Medical University of South Carolina, Charleston, SC 29425, USA

**Keywords:** ovarian cancer, aneuploidy, drug screening, oncology, RAF, genetic vulnerability

## Abstract

Background/Objectives: All 11 metallothionein protein-coding genes are located on human chromosome 16q13. It is unique among human genetics to have an entire pathway’s genes clustered in a short chromosomal region. Since solid tumors, particularly high-grade serous ovarian cancer (HGSC), exhibit high rates of monoallelic aneuploidy, this region is commonly lost. Studies have not yet been performed to determine what vulnerability may be created in cancer cells with low metallothionein expression. Here, a screen of FDA-approved cancer small molecule drugs for those best targeting low metallothionein ovarian cancer was completed. Methods: Screening methods were tested and compared using vehicle-treated negative controls and cadmium chloride, a positive control for cell loss selective for low metallothionein cells. CAOV3 cells, which are unique in their expression of only two metallothionein isoforms, were used, with or without shRNA knockdown of the predominantly expressed *MT2A* gene. A library of FDA-approved molecules was then screened. Results: The optimal assay utilized Hoechst 33342 nuclear staining and mechanized fluorescent microscope counting of cell content. Encorafenib, an RAF inhibitor, was identified as the most selective for enhanced cytotoxicity in *MT2A* knockdown cells compared to scrambled controls. Conclusions: The nuclear stain Hoechst 33342, assessed by fluorescence microscopy, provides a low variance, moderate throughput platform for cancer cell loss screens. Low metallothionein ovarian cancer cells exhibit a vulnerability to the RAF inhibitor encorafenib.

## 1. Introduction

Metallothionein genes are unique among human genetics. Of over 4000 molecular pathways characterized and assessed with more than 10 genes, the metallothionein pathway is the only pathway in which every gene is encoded on the same chromosome arm [1]. This chromosome linkage may be uniquely informative for cancer genetics; normal cells undergo senescence or apoptosis with a disrupted chromosome, but p53 mutation, among others, allows for deadly solid tumors to accumulate aneuploid chromosome losses or gains [2,3,4]. A single chromosome loss would reduce the expression capacity of all metallothionein genes within a tumor, with an unclear biological result.

Metallothionein proteins are small, cysteine-rich proteins. Metallothionein genes are closely related in nucleic acid sequence and amino acid products [5]. There are 11 protein coding metallothionein genes, including 8 *MT1* genes and *MT2A*, *MT3*, and *MT4* isoforms. *MT3* is an isoform more commonly expressed in the brain and *MT4* is more common in the skin. However, the *MT1* and *MT2A* isoforms are the most expressed and distributed throughout the tissues and organs [6,7]. Metallothioneins are cysteine rich, allowing them to bind metal and prevent heavy metal toxicity [8]. Metallothioneins sequester divalent cations, serving as a major repository of intracellular Zn^2+^ ions [9]. When cells are exposed to toxic heavy metals, such as cadmium, copper, mercury, chromium, and arsenic, the heavy metal displaces the chelated Zn^2+^ and induces a conformational change in zinc binding proteins, particularly in metallothioneins [10]. Metallothioneins sequester cadmium [11], preventing the toxic metal from causing damage to the cell. Metallothioneins may interact with oncogenesis through zinc regulation, as low dietary zinc is a risk factor for developing multiple cancers; however, this regulation is likely complex as zinc supplementation is also oncogenic [12]. Understanding the details of metallothionein signaling will be important for contextualizing these opposing etiology signals.

Molecular signaling is complex upstream and downstream of metallothioneins, particularly in cancer. Zinc binds approximately 10% of the proteome [13], so zinc repository control by metallothioneins affects a wide variety of processes. Zinc, alongside induced metallothionein expression, regulates cell biology dependent on the tissue and context, including tumor signaling [14]. Dozens of histologic assessments have sporadically implicated high expression of metallothionein is associated with advanced cancer [15], although it is unclear if any specific metallothionein antibody exists with genetic validation. Metallothioneins are expressed upon MTF1 transcription factor phosphorylation, which increases activity downstream of protein kinase C, casein kinase, and tyrosine kinases [16]. However, metallothionein gene loss is frequent in cancer, and our laboratory has discovered deletions to be associated with elevated aneuploidy and worse prognosis in p53 mutant endometrial cancer [17]. We further found that metallothionein genetic suppression yielded increased DNA damage as assessed by γH2AX foci [1], which is consistent with metallothioneins as tumor suppressors involved in the DNA damage response. The roles of metallothioneins in cell signaling and cancer remain under investigation, but given frequent loss of the gene cluster, exploring low metallothionein levels as a targetable vulnerability is of interest in oncology. Here, ovarian cancer, a deadly cancer with over 19,000 cases and 12,000 deaths predicted in 2025 (National Cancer Institute, Cancer Stat Facts), is chosen as a model due to common loss of metallothionein genes.

Single-enzyme- or kinase-targeted cancer therapies result in evasion of therapy through mutation of the target or through hyper activation of a compensatory pathway [18,19]. Aneuploid pathways, particularly with lost chromosomes, may be attractive complementary targets due to the multiple genes involved. Given the unique opportunity of the metallothionein gene cluster, our laboratory set out to discover targetable biology affected by metallothioneins through drug screening techniques. Following a careful selection of an ideal screening protocol through quality control tests, we discovered that RAF targeting may be uniquely vulnerable for low metallothionein ovarian cancer cells.

## 2. Materials and Methods

### 2.1. Cell Line, Plasmids, Culturing Conditions, and Standard Plastics

Live cells were kept in a 37 °C air-jacketed tissue culture incubator (VWR, #10810-902, Radnor, PA, USA) with 5% CO_2_. All assays utilized RPMI-1640 1× with L-Glutamine media (Corning, #10040CV, Glendale, AZ, USA) supplemented with 10% fetal bovine serum (Gibco, #A5256701, Waltham, MA, USA), 1% sodium pyruvate (Sigma-Aldrich, #S8636, St. Louis, MO, USA), and 1% penicillin streptomycin (Gibco, #15140122). CAOV3 cells (ATCC, #HTB-75, Manassas, VA, USA) were transduced with either shScr (pLKO.1, Addgene #1864, Watertown, MA, USA), shMT2A-1 (TRCN0000148975, Sigma-Aldrich, targeting sequence GCAAAGAGTGCAAATGCACTT), or shMT2A-2 (TRCN0000148783, Sigma-Aldrich, targeting sequence CTGCAAATGCAAAGAGTGCAA). CAOV3 cells were derived from the ovary of a 54-year-old female ovarian cancer patient and are morphologically epithelial. Reverse-transcriptase quantitative polymerase chain reaction (RT-qPCR) was used to validate knockdown, as per our previously published method (primers were TBP F:ACTCCACTGTATCCCTCCCC, TBP R:CCAGAACTCTCCGAAGCTGG, MT2A F:CCCGCTCCCAGATGTAAAGA, MT2A R:TATAGCAAACGGTCACGGTCA) [1]. Fluorescent protein nls-GFP (Addgene, #126688) or nls-BFP (Addgene, #36085) was stably added via lentivirus using third generation lentiviral packaging vectors (Addgene, #12251, 12253, 12259) packaged in HEK293T cells (ATCC, #CRL-3216). Unless otherwise noted, assays were completed in 96-well transparent tissue-culture treated plates (VWR, #10062-900). Cells were seeded to 625–1250 live cells per well using a BioRad TC20 cell counter to assess cell density with trypan blue (Sigma-Aldrich, #T8154-100ML) as a dead cell stain. Culture volume was 100–160 µL. Cells were allowed to adhere and proliferate 24 h prior to addition of treatments. Multi-channel pipettes and tips were tested from a range of manufacturers and distributors including VWR, Corning, and USA Scientific, and the most consistent cell seeding and drug additions were achieved using the USA Scientific ErgoOne^®^ 8- or 12-channel pipettes using 200 µL (USA Scientific, #1120-8810, Ocala, FL, USA) or 300 µL graduated filter tips (USA Scientific, #1120-9810). Further optimization identified that adding 50 µL phosphate buffer saline (PBS) (Corning, #21-040-CV) into each inter-well space in 96-well plates maintained the most consistent humidification and minimized inter-well variation for identical conditions.

### 2.2. Drug and Cadmium Sources

Cadmium chloride (99.99% purity) was procured from Sigma-Aldrich, #202908-10G. Screening drugs were obtained from the National Cancer Institute Developmental Therapeutics Program. Retested uramustine/uracil mustard was ordered from Millipore Sigma (Burlington, MA, USA), #S375063-250MG and encorafenib from MedChemExpress (Monmouth Junction, NJ, USA), #50-149-4034. Dimethyl sulfoxide (DMSO) was ordered from Millipore Sigma, #D8418-100ML. N-acetyl cysteine was from AmBeed, #A233740 (Arlington Heights, Il, USA).

### 2.3. CellTracker™ Green Assay

On day of cell dye loading, CellTracker™ Green CMFDA (ThermoFisher, #C7025, Waltham, MA, USA) was diluted to 10 mM according to manufacturer’s protocol. Serum free media used was Opti-MEM (Gibco, #31985070). All reagents and pre-labeled plastics were warmed to 37 °C prior to protocol initiation. Working solutions of CellTracker™ Green at 0.01 µM, 0.05 µM, 0.1 µM, 0.25 µM, 0.5 µM, 1 µM, 5 µM, and 10 µM were made in Opti-MEM through serial dilutions. Cells were prepared by trypsinization, PBS wash, and resuspension in working solution to seed 1250 per well in these working solutions at 15 min, 30 min, and 45 min loading time increments. After these loading times, each time increment was individually processed. Cells were spun 1000 g for 2 min, resuspended in complete normal media, and then plated into recipient 96-well plates. Cells were allowed to adhere and acclimate for 24 h prior to CdCl_2_ addition. CellTracker™ Green was counted by GFP channel area detected using Gen5 software (V3.11) after acquisition and deconvolution using a 4× objective on an Agilent BioTek Lionheart FX.

### 2.4. CellTiter-GLO^®^ Assay

On day of cell dye loading, CellTiter-GLO^®^ reagents (Promega, #G7570, Madison, WI, USA) were prepared as per manufacturer’s instructions. All reagents were warmed to room temperature prior to cell plate addition. Equal volumes of PBS and manufacturer’s reagents were mixed and 30 µL of this diluted mix was added to each well. Plates were shaken on a rotary mixer at 60 rpm for 10 min and read on a Promega GloMax luminometer at 1000 ms/well.

### 2.5. Nuclear Localized GFP and BFP Nuclei Counting

CAOV3 cells were lentivirally modified with nuclear localization signal (nls) GFP or BFP (see vectors section). GFP or BFP modified cells were sorted to fluorescent purity via flow cytometry prior to assays. GFP and/or DAPI channels were used with a 4× objective on an Agilent BioTek Lionheart FX microscope for image acquisition. Gen5 software (V3.11) was then used to count separate nuclei.

### 2.6. Crystal Violet Viability Assay

The crystal violet (Sigma-Alrich, #61135-100G) staining procedure utilized a modified procedure related to our previous assays [1,20]. A modification to increase throughput without adding to noise nor diminishing signal to noise was to follow these steps: (a) aspirate media, (b) add 50 µL crystal violet fix and stain solution (0.11% crystal violet, 0.17 M NaCl and 22% methanol in ddH_2_O), (c) aspirate, wash with 150 µL PBS, aspirate, (d) dry 37 °C for 30 min, (e) add 50 µL 10% acetic acid (replaces methanol in other protocols), (f) allow 5 min of 60 rpm rotary mix, and (g) read absorbance at 600 nm.

### 2.7. Hoechst 33342 Nuclear Count Viability Assay

Fixation and staining solution consisted of 10 mL 32% paraformaldehyde (PFA) (Electron Microscopy Sciences, #15714, Hatfield, PA, USA) with 8 µL Hoechst 33342 (Invitrogen, #H3570, Waltham, MA, USA). Cell culture volume was 160 µL and 10 µL of this fixation solution was added per well (1.88% PFA final concentration). Fixation was allowed to proceed at room temperature, with 2 min of 60 rpm rotary mix, prior to imaging. Plates were stored in light-covered cabinets in sealed Ziploc bags for assessments of time-delayed imaging. A mechanized fluorescent microscope (Agilent Biotek, #Lionheart FX, Santa Clara, CA, USA) using a 1.25× objective under the DAPI channel was used to image nuclei, with analysis of nuclei counts on Gen5 software (V3.11).

### 2.8. Drug Screen of an FDA-Approved Drug in Cancer Indication

Cells were seeded into 96-well tissue-culture treated plates (VWR, #10062-900) containing inter-well 50 µL PBS at a density of 625 cells per 50 µL complete RPMI. Immediately after adding cells to plate, the plate was tapped gently against a biosafety cabinet hood twice to foster cell dispersion across the well. Twenty-four hours later, 110 µL complete media was added to all wells with varying levels of test drug concentration. A dilution series of 2.5 µM, 0.5 µM, and 0.1 µM test drug was added to each plate, with each plate containing the same drugs tested on both shScr and shMT2A cells in a mirrored layout. DMSO concentration range was 0.31%. Cadmium chloride (Sigma-Aldrich, #202908) was diluted in molecular biology grade water (VWR, #L0201-0500) to 2 mM and added to each screen plate at 30 µM, 20 µM, and 10 µM as an internal control. Then, 72 h after drug addition, Hoechst 33342 PFA fixing and staining solution (see above) was added to each well in the plate at room temperature. Plates were rotated at low speed for 2 min for fixation and staining. Longer mixing was avoided to prevent cell disruption. Plates were then imaged on a mechanized fluorescent microscope (Agilent Biotek, #Lionheart FX) using a 1.25× objective under the DAPI channel or a robotic linked fluorescent microscope (Agilent Biotek, #Cytation 5) using a 2.74× objective under the DAPI channel. Nuclei number per well were quantified using Gen5 software (V3.11). Cell loss (%) was calculated as (1 − [# nuclei, no drug]/[# nuclei, drug]) ∗ 100. Two–three experimental replicates were performed for each drug candidate. The Area Under the Curve (AUC) metric summed all cell loss values and the shScr AUC was subtracted from the shMT2A AUC to derive the selectivity AUC metric.

### 2.9. Western Immunoblots 

Cells were seeded on 10 cm dishes at a density of 600–800 k cells. Cells were treated with vehicle control (equi-volume DMSO and water, which were diluents for encorafenib and NAC, respectively), 500 nM encorafenib, or 500 nM encorafenib with 1 mM NAC. After 48 h, cells were aspirated of media, rinsed once with 10 mL PBS, and lysed in 400 µL RIPA buffer (10 mM Tris-HCl pH 8.0, 1 mM EDTA pH 7.5, 1% Triton X-100, 0.1% sodium deoxycholate, 0.1% sodium dodecyl-sulfate, 140 mM sodium chloride). Lysates were normalized to 1 µg/µL, diluted with 6× reducing laemmli sample buffer (Thermo Scientific, #J61337.AD), and boiled for 5 min at 95 °C prior to loading on SDS-PAGE gels as previously described [21]. Primary antibodies were Phospho-c-MYC Ser62 (E1J4K, Cell Signaling #13748, Danvers, MA, USA), c-MYC (D84C12, Cell Signaling #5605), Phospho-p44/42 MAPK ERK1/2 Thr202/Tyr204 (20G11, Cell Signaling #4376), ERK2 antibody (1B3B9, gift from Michael J. Weber, Charlottesville, VA, USA), and Vinculin (V284, Santa Cruz Biotechnology #59803, Dallas, TX, USA).

### 2.10. Morphological Brightfield Microscopy

Cells were seeded and treated as in Western immunoblot section. At 42 h, cells were imaged on a BioRad ZOE microscope using a 10× objective. Morphology was quantitatively assessed using CellProfiler [22] version 4.2.8: cells were segmented and lengths and widths calculated. Scrambled control cells were set to a normalized level of 1 for the elongation metric, with other conditions compared relative to this normalized value to enable rational comparison. Equal numbers of cells were assessed per condition, using 100 cells per image in each of two independent experiments, as equalized via FairSubset software (V1.0) [23].

## 3. Results

### 3.1. Genetics of Metallothioneins in Ovarian Cancer

**Figure 1:**
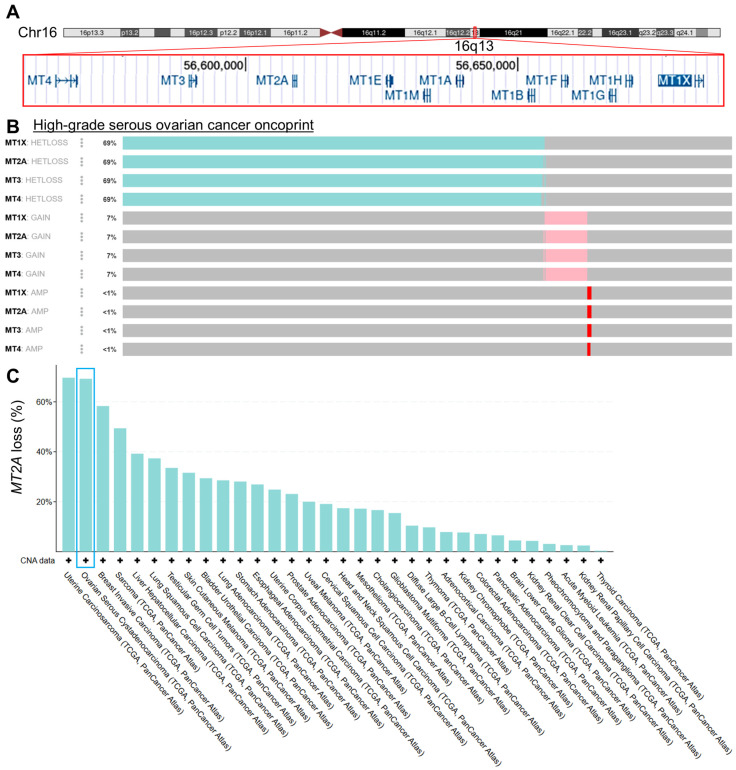
Metallothionein genetics in cancer. (**A**) University of California Santa Cruz Genome Browser view of the metallothionein cluster. All 11 protein-coding metallothionein genes are encoded on Chr16q13, with no intervening protein-coding genes. (**B**) Metallothioneins 1–4, with *MT1X* as representative for the distal *MT1* gene, were queried for loss, amplification, and gain events in high-grade serous ovarian carcinoma in The Cancer Genome Atlas Pan-Can dataset in samples with copy-number alteration (CNA) data (N = 572 tumors). An oncoprint is shown, with each column representing a single patient. Non-synonymous mutations were not observed (0% of samples for these genes) (**C**) *MT2A* loss was queried across the PanCancer Atlas tumor dataset, for samples with CNA data. Ovarian cancer, the focus of this study, is outlined in cyan.

Metallothionein-protein-coding genes are all located on human chromosome region 16q13 (Figure 1A). There are 11 genes in total. No non-metallothionein protein coding genes exist between the metallothioneins within this gene cluster. High-grade serous ovarian cancer (HGSC) was queried for heterozygous loss (HETLOSS), single-allele gain (GAIN), or multiple allele amplification (AMP) of four metallothionein genes within this cluster, using cBioPortal maintained pan-cancer The Cancer Genome Atlas (TCGA) data [24,25,26]. Losses (69%) heavily outnumbered gains (7%), with nearly identical numbers for each metallothionein gene (Figure 1B). Homozygous deletions were rare (0.35%). Given their close linkage, similar event counts were expected. Zero non-synonymous single-nucleotide variant or indel mutations in these genes were found, indicating gene mutation changes rarely, if ever, drive metallothionein biology in cancer. As a representative gene, *MT2A* was queried across pan-cancer TCGA tumors in different tumor types (Figure 1C). HGSC was second only to the rare uterine carcinosarcoma for metallothionein gene loss percentage. Most solid cancers exhibited *MT2A* gene loss in over 10% of cases.

### 3.2. Metallothionein Controlled Zinc and Cadmium Toxicity

**Figure 2:**
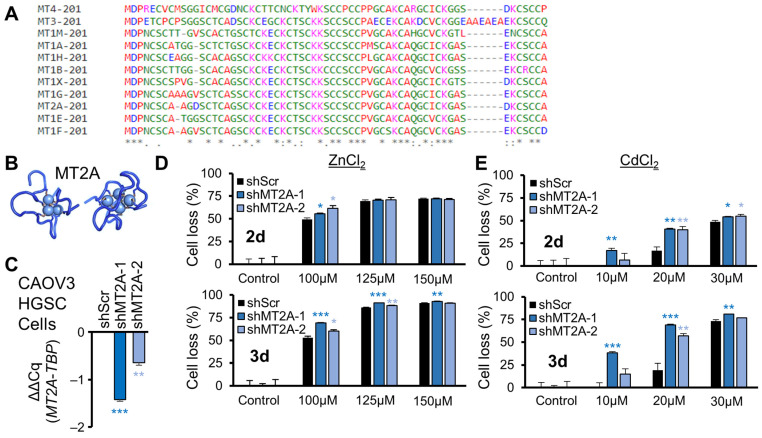
Zinc and cadmium metal toxicity in *MT2A* knockdown. (**A**) Amino acid alignment of the 11 expressed human metallothioneins. Note that cysteine residues, which chelate metals, are highly conserved among the proteins and represent a third of all the amino acids in metallothioneins. (**B**) Nuclear magnetic resonance structure of metallothionein MT2A alpha (PDB ID: 2MRB) and beta (PDB ID:1MRB) domains, with bound Cd^2+^ ions shown. Zn^2+^ ions are predicted to be similarly located within the protein in the absence of Cd^2+^. (**C**) CAOV3 cells, which only express *MT2A* (higher) and *MT1X* (lower), were knocked down by lentiviral shMT2A and validated by RT-qPCR as reduced in expression relative to scrambled shRNA control (shScr). (**D**) ZnCl_2_ toxicity was assessed by Hoechst-33342-stained nuclei counts relative to control treated cells, with 2-day (2 d) or 3 d exposure. (**E**) CdCl_2_ toxicity was assessed as in (**D**). Error bars are standard error of the mean. * *p* < 0.05, ** *p* < 0.01, *** *p* < 0.001 by *t*-test of technical replicates relative to shScr control from a representative experiment.

To construct and quality control an assay to find drugs selective for low metallothionein cancer cell line toxicity, a positive control was needed. Metallothionein proteins are highly similar at the amino acid level (Figure 2A) due to their conserved role in zinc chelation [8,9]. In the presence of heavy metals, including cadmium, zinc is displaced within metallothioneins [10,11]. The alpha domain contains three ion binding sites and the beta domain contains four ion binding sites, encompassing most of the internal volume of metallothioneins (Figure 2B). To create a well-controlled screen, ovarian cancer cells were engineered for variable suppression of MT2A relative to a control. CAOV3 HGSC cells were selected, due to uniquely restricted expression among all ovarian cancer cell lines of only *MT2A* and *MT1X*, with *MT2A* as the predominantly expressed metallothionein [1,27]. Short-hairpin RNAs (shRNAs) targeting MT2A were used compared to a scrambled shRNA control (shScr) to suppress MT2A mRNA, as assessed by RT-qPCR (Figure 2C). To assess which control may be more selective for low MT2A cytotoxicity, viability assays were performed with ZnCl_2_ and CdCl_2_ insults. Zinc chloride was modestly selective in increasing shMT2A cell loss (Figure 2D), while cadmium chloride was consistently selective in increasing shMT2A cell loss relative to shScr across a broad range of cadmium concentration (Figure 2E). Cadmium selectivity was more apparent with 3 days of metal exposure than 2 days, so viability assays throughout this study were conducted using cadmium as a positive control with 3 days of exposure. 

### 3.3. Assessment of Screening Methods

To determine a minimally variable, moderate throughput assay to assess compounds selective for increased shMT2A cytotoxicity relative to shScr cells, commonly utilized methods of cell counting were tested. In a screen, the shMT2A-1 line is predicted to yield the largest signal to noise relative to shScr and was used in the quality control selection of assays.

#### 3.3.1. Live Cell Methods

**Figure 3:**
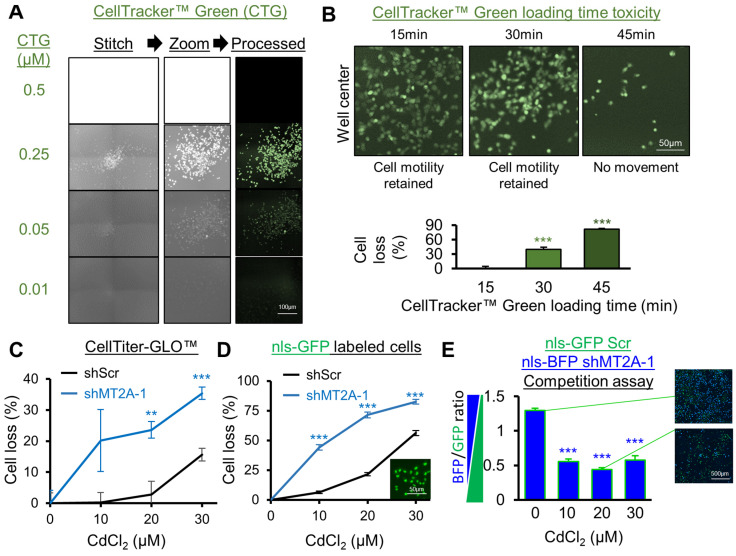
Comparison of live cell viability assays for sensitive detection of selectivity. For all panels, CAOV3 cells were assessed with the indicated shRNAs or by shScr if not indicated. (**A**) CellTracker™ Green, a common fluorescent cell labeling method in screens, was evaluated for a range of loading concentrations for imaging sensitivity and specificity. (**B**) CellTracker™ Green was evaluated for length of dye loading time and resulting toxicity, related to panel (**A**). Migration toward the center of the well over a period of 72 h post-loading was assessed as a toxicity phenotype. Cell loss via CellTiter-GLO™ was measured at the end of 72 h for the variable CellTracker™ Green loading times. (**C**) CellTiter-GLO™, a common luminescent plate reader screening assay, was utilized following 72 h of CdCl_2_ treatment. (**D**) Cells were lentivirally labeled with nuclear localized (nls) GFP and measured for cell counts via fluorescent microscopy following 72 h of CdCl_2_ treatment. A representative image of nls-GFP labeled cells is shown (inset). (**E**) CAOV3 shScr cells were lentivirally labeled and flow-cytometry sorted for nls-GFP, whereas CAOV3 shMT2A-1 cells were similarly labeled with nls-BFP. Each were seeded at equal cell numbers into recipient plates for a 72 h competition assay with varying CdCl_2_ levels. Fluorescent microscopy was used to calculate nuclei count for each cell line and the ratio is quantified here. Representative images are highlighted for 0 and 30 μM CdCl_2_. Error bars are standard error of the mean. ** *p* < 0.01, *** *p* < 0.001 by *t*-test of technical replicates relative to control from a representative experiment.

Assays utilizing live cells can be attractive as the measures of viability require active cell metabolism. One well-cited example (N = 102 PubMed indexed articles) is the CellTracker™ dye system, which relies on cytoplasmic glutathione transferase activity to convert a reactant into a cell-impermeable fluorescent molecule detectable by plate readers and microscopy. Once converted, the dye is retained in the cell for multiple cell divisions. Using manufacturer recommendations, a range of CellTracker™ Green CMFDA cell loading concentrations (Figure 3A) and loading times (Figure 3B) were tested. A narrow concentration range for dynamic imaging was observed, peaking at 250 nM. Loading toxicity was severe at loading incubation times longer than 15 min. This is possibly due to a requirement for low serum medium during the loading period, or due to dye toxicity in this loading period.

Another common live-cell viability assay, due to its high signal to noise ratio in a plate reader setting, is CellTiter-GLO^®^ (N = 285 PubMed indexed articles). Live cells are required due to assay utilization of luciferin by luciferase, which, using cellular ATP, converts the reactant to oxyluciferin light-producing product. Using this assay, high selectivity was observed for shMT2A cadmium toxicity relative to shScr cells, although the magnitude of cell loss detected was not quite as high as the initial cadmium toxicity assays and high variation, dependent on well location, was observed (Figure 3C). This is partly due to transparent plate utilization, which is inexpensive, but raises cross-well contamination of signal for plate-reader-based assays.

Next, cells were lentivirally labeled by the nuclear localized fluorescent protein nls-GFP and purified for presence of fluorescence by flow cytometry. In this assessment, shScr and shMT2A cells were independently labeled by nls-GFP. Cells were then treated with CdCl_2_ for 72 h and then nuclei were counted by live-cell microscopy using a 37 °C 5% CO_2_ chamber on a mechanized fluorescent microscope using the GFP channel. Cell loss was readily apparent with high magnitude and high selectivity for an increase in loss in shMT2A cells relative to separately assessed shScr cells (Figure 3D). This was somewhat surprising, as phenol red is present as a pH indicator in standard media and can give rise to background signal in the GFP channel. Phenol red was retained in this assay, yet it did not limit sensitive cell loss detection.

A competition assay using both shMT2A and shScr cells in the same well was next evaluated. In this case, lentiviral nls-GFP labeled shScr cells and nls-BFP labeled shMT2A cells were independently sorted by flow cytometry for their respective fluorescence. The competition assay utilized equal seeding of both cells, which were then treated with 0–30 µM CdCl_2_ and assessed for relative counts 72 h after cadmium exposure. Due to imaging acquisition and processing nuances wherein BFP yielded less diffuse image results than GFP, untreated cells were counted with BFP as slightly more prevalent than GFP cells. However, CdCl_2_ changed the ratio to favor GFP (shScr) cell counts at each dose tested, consistently demonstrating selective cytotoxicity toward shMT2A cells (Figure 3E).

#### 3.3.2. Fixed Cell Methods

**Figure 4:**
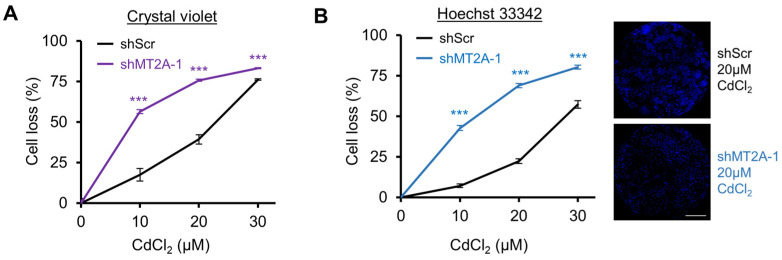
Comparison of fixed cell viability assays for sensitive detection of selectivity. For all panels, CAOV3 cells were assessed with the indicated shRNAs. (**A**) Crystal violet viability assay following 72 h CdCl_2_ exposure. (**B**) Hoechst 33342 microscopic nuclei counting assay of fixed cells following 72 h CdCl_2_ exposure. Representative, visually optimized whole-well stitched images are shown. Scale bar is 1 mm. Error bars are the standard error of the mean. *** *p* < 0.001 by *t*-test of technical replicates relative to control from a representative experiment.

Assays utilizing fixed cells can minimize some variation attributed to nuances in processing live cells; once fixed, the cells no longer change, allowing for more flexibility in plate reads. Many fixed cell assays exist. Here, we describe crystal violet staining (N = 993 PubMed indexed articles) and Hoechst 33342 nuclei staining (uncommon as a viability assay) as the methods we found to yield strong differences between the genetically altered cell lines with minimal variation. Crystal violet stains negatively charged biomolecules, including proteins and nucleic acids [28]. Crystal violet staining procedures have wash steps which rinse dead cells off the plate, so only adherent cells remain in the quantification step. Crystal violet is solubilized using methanol or, in these presented assays, 10% acetic acid, which was important for the laboratory these procedures were performed in due to high humidity confounding methanol-based resuspension. Absorbance then detects crystal violet levels. Like the live cell assays, crystal violet was capable of demonstrating strong separation of shMT2A and shScr dose–response viability curves when exposed to cadmium cytotoxicity (Figure 4A). The magnitude of cell loss was higher than CellTiter-GLO^®^, possibly due to the wash step, and variation was lower. However, it was observed that first-time users of this assay inadvertently drip crystal violet from the edge of pipette tips into wells, providing abnormally high absorbance values uncorrelated to cell density. Thus, it is not a robust assay for many users. 

Hoechst 33342 staining, in contrast, resulted in reproducible results among multiple users. The protocol was optimized to have a single liquid transfer step for dual fixation and staining. Hoechst 33342 is membrane permeant, binding to DNA in live or dead cells [29]. With fluorescent imaging, individual nuclei are apparent even in confluent areas of cell culture. This method resulted in the least amount of variation between identically treated samples, providing a clear difference in CdCl_2_ lethality between shScr and shMT2A cells (Figure 4B). Since Hoechst 33342 can stain live or dead cells, cells were fixed, and the interpretation is cell count changes over a period of growth.

#### 3.3.3. Summary of Methods and Assay Choice

The datasets yielded information on the repeatability, variation, and cost of each assay. The scalability was assessed for these assays taking into account all technical aspects of the protocol experienced during assay development. These characteristics are summarized in Table 1.

To screen a compound collection, the highest value was placed on lowest standard deviation between identically treated wells. Altogether, the nuclear Hoechst 33342 staining and microscopic counting yielded the highest screen value of the six methodologies tested. Using 20 µM CdCl_2_ treated shMT2A wells compared to 20 µM CdCl_2_ shScr wells, a Z factor (Z’) of 0.69 was calculated [30]. This low variation value was sufficient to move forward with compound screening.

#### 3.3.4. Robotic Method

**Figure 5:**
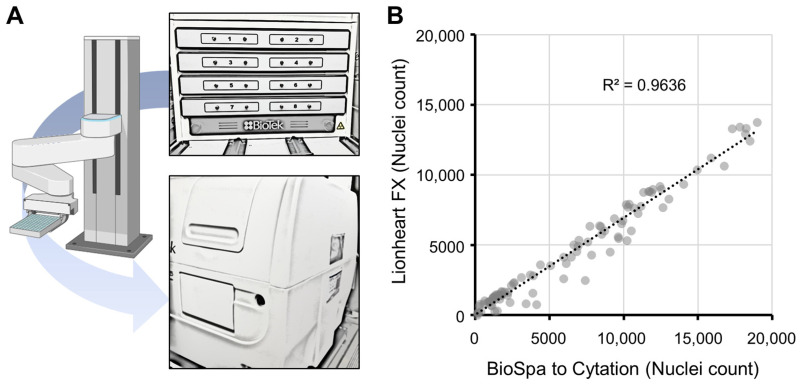
Robotic processing of Hoechst 33342 viability assays. CAOV3 shScr cells were assessed following 72 h exposure of 12 drug candidates in a 3-level dose response. (**A**) Schematic of robotic workflow with fluorescent Cytation microscope and BioSpa plate holder (up to 8-plate runs). (**B**) Hoechst 33342 microscopic nuclei counting assay via Lionheart FX microscope compared to Cytation-BioSpa pair. A linear trend line between the two assays was calculated and shown.

The Hoechst-33342-stained cells were assessed on a mechanized fluorescent microscope (Agilent BioTek Lionheart FX) similar in character to a robotic-capable microscope (Agilent BioTek Cytation 5, linked with robotic plate changing BioSpa) (Figure 5A). To understand if the two reading modalities were similar in data output despite changes in hardware, each was assessed using an identical plate with varying cell density. A high R-squared value was obtained, indicating that despite different hardware and analysis settings, the Hoechst 33342 relative results were similar (Figure 5B). It is notable that overall cell counts differed due to technical nuances associated with plate well areas analyzed by Gen5 software. Since the goal was to find relative differences in shScr versus shMT2A cells rather than absolute cell counts, either read modality was found to be appropriate for screening. 

### 3.4. Screen of FDA-Approved Cancer Drugs for Low Metallothionein Selectivity

**Figure 6:**
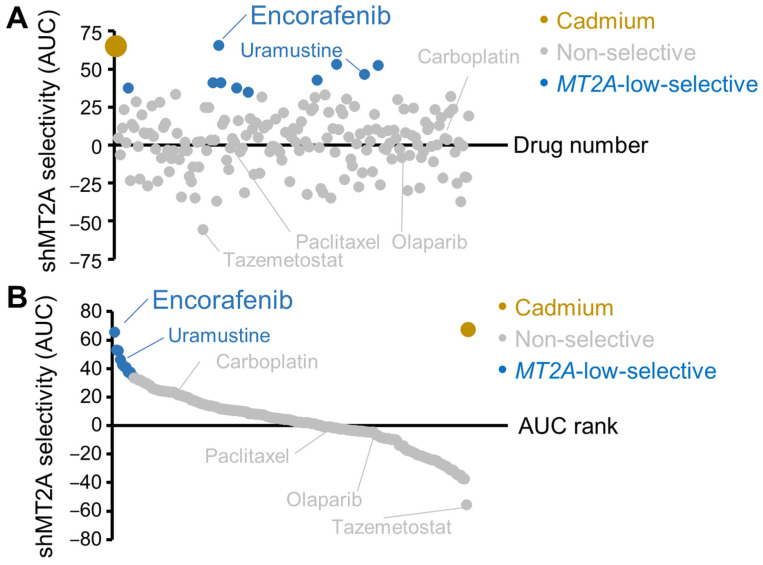
FDA-approved cancer drug screen. CAOV3 ovarian cancer cells were assessed. Results utilized 3-day drug exposure and fixed cell Hoechst 33342 nuclei counting on the Lionheart FX. Area under the curve (AUC) of cell loss from shMT2A-1 with shScr subtracted yielded the selectivity metric, with a positive number indicating more shMT2A-1 cell loss than shScr. (**A**) Range of all drugs (N = 179 small molecules) tested. The threshold for *MT2A*-low selective compounds was two standard deviations above the mean of all drugs. Carboplatin, paclitaxel, and olaparib are highlighted due to their common use in high-grade serous ovarian cancer treatment. (**B**) A ranked representation of the data presented in panel (**A**). In both panels, AUC of CdCl_2_ is shown for positive control comparison.

To determine what existing FDA-approved cancer drugs, many of which remain in use for solid tumor treatment, may most impact ovarian cancers with low metallothionein expression, a chemical library was obtained from the National Cancer Institute (see methods). This library was screened at three doses, i.e., 2.5 µM, 0.5 µM, and 100 nM, with each plate containing untreated control cells to enable a calculation of relative cell loss. CAOV3 cells with either shScr or shMT2A were arrayed with each cell line on the same plate in varying rows, with each drug tested with this dilution series in columns. Plates were incubated with drugs or control CdCl_2_ for 72 h. Plates were then fixed and stained by Hoechst 33342 and counted by fluorescence microscopy. Since the goal was to find drugs most effective at incurring cell loss in shMT2A relative to shScr, an area-under-the-curve (AUC) metric subtracting shScr loss from shMT2A loss was used across all doses tested. Results were averaged between the 2–3 assays performed for each drug and tabulated. There did not appear to be a plate order bias (Figure 6A, plotted by physical drug order), as hits were distributed throughout the chemicals tested. Ranked plotting revealed a small handful of the 179 drugs tested were more than 2 standard deviations different from the mean of all drugs tested (Figure 6B). Outliers which were selective in killing shMT2A more than shScr cells included encorafenib, an RAF inhibitor, and uramustine/uracil mustard, a DNA alkylating agent. While they did not meet the threshold criteria, other RAF inhibitors, i.e., vemurafenib, debrafenib, and sorafenib, trended for shMT2A selective cell loss, as did the downstream kinase MEK1/2 inhibitor trametinib (Supplementary Table S1). Other chemotherapies used in HGSC, including carboplatin, paclitaxel, and olaparib, were not selective for MT2A levels. An outlier uniquely non-sensitive to shMT2A cells was tazemotostat, an epigenetic regulator through EZH2 methyltransferase inhibition [31].

### 3.5. Encorafenib Selectivity Targets Engineered Low Metallothionein Ovarian Cancer

**Figure 7:**
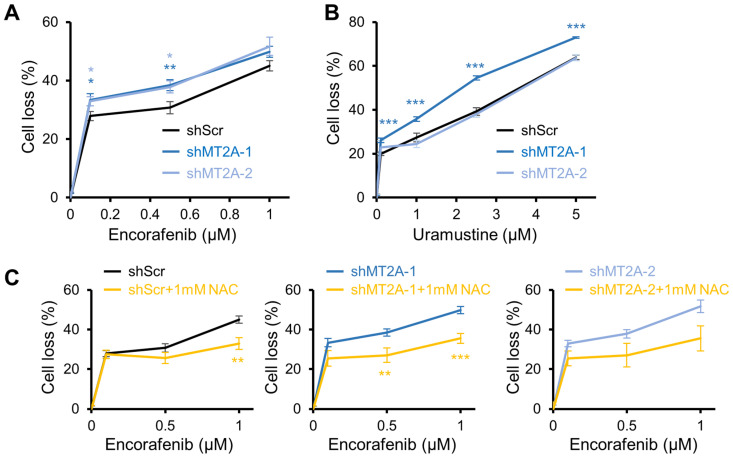
Screen validation confirms encorafenib selectivity in MT2A-low cells. CAOV3 ovarian cancer cells with the indicated shRNAs were assessed at 72 h of drug exposure. (**A**) The BRAF inhibitor encorafenib was independently purchased, diluted, and assessed through the Hoechst 33342 viability assay. (**B**) The alkylating agent uramustine/uracil mustard was independently purchased, diluted, and assessed through the Hoechst 33342 viability assay. (**C**) Mechanism of encorafenib selectivity was assessed through the concurrent addition of reactive oxygen species scavenger N-acetyl cysteine (NAC). The mean of two experiments (uramustine) or five experiments (encorafenib) are displayed. Error bars are standard error of the mean. * *p* < 0.05, ** *p* < 0.01, *** *p* < 0.001 by *t*-test of technical replicates relative to control from all experiments.

Validation assays were next performed on encorafenib and uramustine, two highly selective hits for elevated shMT2A toxicity relative to shScr. New chemicals were purchased from an independent vendor (see Methods), reconstituted in DMSO, and applied to new dose–response studies. The Hoechst 33342 nuclei counting method was used in the secondary validation. Encorafenib retained its enhanced cytotoxicity in shMT2A-1 as well as the second knockdown shMT2A-2 (Figure 7A). Uramustine, conversely, remained selective in the greater magnitude knockdown shMT2A-1, but was no longer selective in the more moderate knockdown shMT2A-2 cells (Figure 7B), perhaps due to a threshold effect of metallothionein levels. Given the robust recapitulation of shMT2A sensitivity using encorafenib, it was of interest to evaluate the mechanistic connection to metallothioneins. Metallothioneins regulate reactive oxygen species (ROS) through regulation of zinc-finger transcription factors [32], mobilization of zinc [33], and contributing to the redox cycle of glutathione [34], the primary cytoplasmic antioxidant. ROS signals through the BRAF/CRAF-MEK-MAPK pathway, often upstream of RAF through RAS activation [35]. N-acetyl cysteine (NAC) is a ROS scavenger [36], so it was tested for its ability to attenuate ROS-based signaling in the encorafenib dose response. Scavenging ROS with NAC abolished the shMT2A selectivity of encorafenib and reduced cell loss (Figure 7C). 

**Figure 8:**
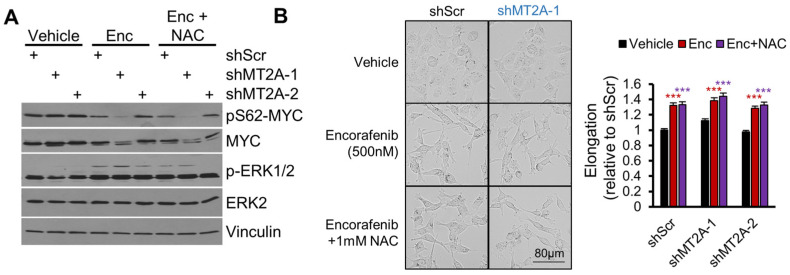
Encorafenib reduces MYC in shMT2A through non-canonical signaling. CAOV3 cells with the indicated shRNAs were assessed for RAF signaling effects. (**A**) Western blots of cells treated with 500 nM encorafenib (Enc), with or without 1 mM N-acetyl cysteine (NAC), with lysates collected at 48 h. Genotype is indicated with “+”. Primary antibodies are labeled. Representative blots from N = 2 experiments. (**B**) Brightfield microscopy of cells treated as in (**A**), imaged at 42 h. Quantitation was mean ± standard error from two experiments, ≥200 cells measured per condition. *** *p* < 0.001 by *t*-test relative to vehicle control.

**Figure 9:**
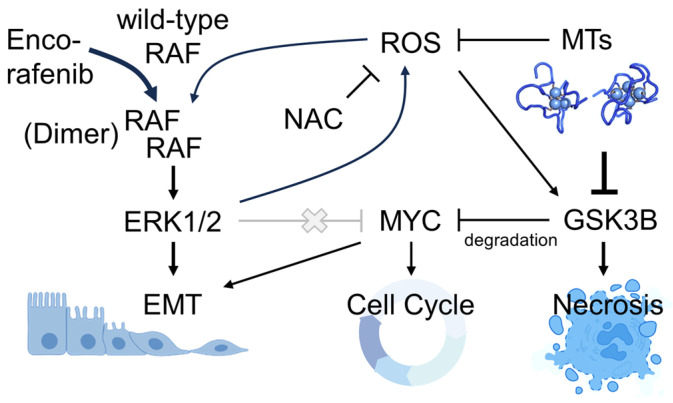
Encorafenib non-canonical RAF signaling model. Model of non-canonical RAF signaling following encorafenib treatment. Thick arrows reflect observed signaling in encorafenib-treated cells with MT2A knockdown.

To evaluate RAF signaling related to encorafenib treatment, immunoblots and morphological microscopy were completed. RAF modulates the epithelial–mesechymal transition (EMT) as well as cell cycle regulatory factors, both of which may be implicated in the enhanced cell loss observed selectively in shMT2A cells relative to shScr cells. Ovarian cancer EMT and proliferation are driven by MYC, which is stabilized from ubiquitin-mediated proteolysis by serine-62 phosphorylation of MYC by active ERK1/2 [37]. Unexpectedly, encorafenib treatment increased phospho-ERK1/2 levels and decreased MYC protein, correlating with reduced phospho-S62-MYC (Figure 8A). MYC decreases following encorafenib were most prominent with shMT2A-1. This effect was independent of NAC treatment. EMT is associated with elongated cells as epithelial cancers lose epithelial identity during the transition. While encorafenib might be predicted to reduce an EMT phenotype, we instead observed elongation to increase with encorafenib treatment (Figure 8B). This effect was again independent of NAC treatment. The data lead to a non-canonical model of cell signaling, in which BRAF inhibition by encorafenib increases phospho-ERK and reduces MYC expression selectively in shMT2A cells, resulting in the observed selective cell loss (Figure 9).

## 4. Discussion

Here, we discovered that targeting RAF, particularly through encorafenib, uniquely causes cell loss for cells engineered with low metallothionein expression compared to otherwise isogenic control cells. To do this, a variety of screening method techniques were quality controlled and compared, yielding our choice of the most consistent assay as nuclear counts using fixation with PFA and staining with Hoechst 33342. Cadmium was more selective in cancer cell toxicity due to low metallothionein expression than zinc was. These results suggest a novel, unexpected connection between metallothioneins and the RAF/MAK/ERK pathway.

Many laboratories have assessed chemicals for cytotoxicity in genetically modified cancer cells, but method comparisons among these assays are rarely present in the literature. In this study, identical cell lines and identical positive controls were used across assays, enabling a quantitative and qualitative comparison of techniques. Plate reader assays exhibited larger variation in general, which is limiting if a large chemical screen of thousands of compounds with just two duplicate test wells of information are available for ranking. Proprietary viability assessment compounds did not outperform other measures available at low cost. While the lowest variation was found through microscopic nuclei counting, this presents a feasibility limitation in some laboratories, as mechanized semi-automated fluorescent microscopy is not always readily available. In these cases, CellTiter-GLO^®^ may be an ideal option, potentially with the reduced material consumption achieved here (15% of vendor recommended reagent volumes yielded sufficient signal/noise), although opaque plates are recommended. Crystal violet is another low-variation plate reader option, but requires development of experienced pipetting technique or robotic liquid transfers to reduce staining artifacts. The current screen was relatively small, but is scalable to thousands of compounds. Additional mechanistic compound screening may yield further insights into metallothionein cell signaling pathways important to cancer cells.

Encorafenib, and the other RAF inhibitors which trended in same direction, may be exciting research agents for metallothioneins and ovarian cancer. CRAF has been implicated as critical to allow for aneuploid cell survival [38,39]. Given the dependency discovered here with low metallothionein cells, this RAF dependence finding is consistent with our previous finding that metallothioneins separate aneuploid-high versus aneuploid-low p53-mutant endometrial cancers [17]. The direct molecular connection remains unclear. Some transcription factors downstream of ERK are zinc finger transcription factors, including MAZ [40] and ZNF580 [41], which may be disrupted by metallothionein levels. Our results indicate reactive oxygen species are involved, as scavenging ROS through N-acetyl cysteine reduced the cell loss from encorafenib and abolished the selective cytotoxicity of encorafenib for low metallothionein cells. Metallothioneins are thought to protect cells against ROS, although the mechanisms are varied and results differ in some studies [42,43]. Indeed, levels of ROS and specific ROS subspecies can lead differentially to RAS/RAF signaling and cancer cell proliferation, or, at high levels, to DNA damage and apoptosis or ferroptosis [44,45].

The mechanism of enhanced cell loss through encorafenib treatment evidently utilizes non-canonical signaling. ERK1/2 signals directly to the commonly amplified oncoprotein MYC via phosphorylation of serine 62 [46]. In this case, reduced metallothionein levels may further activate amplified MYC through the RAF/MEK/ERK signaling pathway. RAF inhibition, through encorafenib, would be expected to reduce phospho-ERK1/2, yet we observed an increase. Others have observed this effect in wild-type BRAF cells due to an activation or priming of RAF through dimerization incurred by RAF inhibitors [47,48], which is consistent with our immunoblot and morphologic data. However, canonical RAF-ERK signaling would lead to stabilization of MYC. We observed the opposite. While further experimentation is necessary to confirm the complete molecular mechanism, destabilization of MYC was likely through ROS signaling activating GSK3β, which causes MYC degradation [37] as well as necrosis [49] (Figure 9). Bypassing this RAF signaling through both downstream effector and parallel pathway signaling may be important for clinical benefit [50]. Low-grade serous ovarian cancers are now often treated with the ERK1/2 inhibitor trametinib due to an improvement compared to previous standard of care [51]. These results may indicate low-grade ovarian cancer patients with 16q13 metallothionein cluster loss may further benefit from trametinib treatments. Pre-clinical studies with controlled metallothionein expression levels in specific cancer types are needed to address such possibilities.

## 5. Conclusions

Low metallothionein ovarian cancer cells exhibit a previously undiscovered vulnerability to the RAF inhibitor encorafenib.

## Figures and Tables

**Table 1:** Summary of features of the screening techniques assessed.

Assay	Value Rank	Standard Deviation (%)	Strengths	Limitations
Hoechst 33342 (fixed)	1	3.2	Lowest standard deviation Consistent among investigators Z-factor up to 0.69 High dynamic range Single pipette step for read assay, no washing Fixed cells can be evaluated later Low cost	Slower per plate read than non-microscopic plate readers
nls-GFP, counts (live)	2	3.9	Low standard deviation High dynamic range No pipette step for read assay, no washing Low cost	Live cells require immediate read Each line must be modified by lentivirus Moderate imaging time
nls-GFP/BFP, competition (live)	3	13.9	High dynamic range No pipette step for read assay, no washing Low cost	High standard deviation Live cells require immediate read Each line must be modified by lentivirus Longer imaging time
Crystal violet (fixed)	4	4.3 *	Low standard deviation * * Dependent on investigator experience level High dynamic range Fixed cells can be evaluated later Fast plate read Low cost	Higher variation with some investigators (easier than other assays to acquire technical artifacts) Wash steps required
CellTiter-GLO^®^ (live)	5	7.9	High dynamic range Fast plate read Single pipette step for read assay, no washing	Moderate standard deviation Live cells require immediate read Moderate cost
CellTracker™ Green (live)	6	25.6	No pipette step for read assay	Highest variation Low dynamic range of loading time and dye doses Requires low-serum incubation and media changes Live cells require immediate read Slower per plate read than non-microscopic plate readers Moderate cost

## Data Availability

The datasets used and/or analyzed during the current study are available herein as tables, figures, and data. Source data are available from the corresponding author upon reasonable request.

## Supplementary Materials

The following supporting information can be downloaded at: https://www.mdpi.com/article/10.3390/genes16010042/s1, Table S1: shMT2A FDA cancer drug screen data.
